# How Our Perception and Confidence Are Altered Using Decision Cues

**DOI:** 10.3389/fnins.2019.01371

**Published:** 2020-01-14

**Authors:** Tiasha Saha Roy, Bapun Giri, Arpita Saha Chowdhury, Satyaki Mazumder, Koel Das

**Affiliations:** ^1^Department of Mathematics and Statistics, Indian Institute of Science Education and Research Kolkata, Mohanpur, India; ^2^Department of Psychology, University of Wisconsin-Milwaukee, Milwaukee, WI, United States; ^3^Department of Anesthesiology, University of Michigan, Ann Arbor, MI, United States

**Keywords:** perceptual decision making, social influence, computational modeling, gamma mixture model, multivariate pattern classification

## Abstract

Understanding how individuals utilize social information while making perceptual decisions and how it affects their decision confidence is crucial in a society. To date, very little has been known about perceptual decision-making in humans and the associated neural mediators under social influence. The present study provides empirical evidence of how individuals are manipulated by others' decisions while performing a face/car identification task. Subjects were significantly influenced by what they perceived as the decisions of other subjects, while the cues, in reality, were manipulated independently from the stimulus. Subjects, in general, tend to increase their decision confidence when their individual decision and the cues coincide, while their confidence decreases when cues conflict with their individual judgments, often leading to reversal of decision. Using a novel statistical model, it was possible to rank subjects based on their propensity to be influenced by cues. This was subsequently corroborated by an analysis of their neural data. Neural time series analysis revealed no significant difference in decision-making using social cues in the early stages, unlike neural expectation studies with predictive cues. Multivariate pattern analysis of neural data alludes to a potential role of the frontal cortex in the later stages of visual processing, which appeared to code the effect of cues on perceptual decision-making. Specifically, the medial frontal cortex seems to play a role in facilitating perceptual decision preceded by conflicting cues.

## 1. Introduction

In today's information-satiated society, perceptual decision and subsequent action are greatly influenced by social information. Modern human society is increasingly organized around collective opinions, as reflected in people's increased use of web ratings for daily choices about consumer products, lodging, food, and entertainment (Jayles et al., 2017). Opinions and choice can easily propagate through social networks (Jansen et al., 2009; Gonçalves and Perra, 2015) in this digitized world, and even political opinions can be manipulated using social transmission (Bond et al., 2012). The human tendency to conform to social influence has been explored systematically in classic studies by Solomon Asch (Asch and Guetzkow, 1951; Asch, 1955) and others (Berns et al., 2004, 2010; Behrens et al., 2008; Klucharev et al., 2008, 2009, 2011; Campbell-Meiklejohn et al., 2010; Biele et al., 2011; Izuma and Adolphs, 2013 and see Tajfel, 1982; Cialdini and Goldstein, 2004; Izuma, 2013 for reviews). Reliance on other's opinion is not unique to humans. Different species of animals depend on collective opinion to decide on life-critical perceptual tasks like foraging for food, placement of nests and navigation (Simons, 2004; Conradt and List, 2009; Couzin, 2009) and evolve optimal decision-making strategies accordingly. Consideration of the beneficial effect of group decision can be traced back as early as 1907, when Francis Galton analyzed the opinions of 787 people about the weight of an ox and found that combining their numerical assessments resulted in a median estimate that was remarkably close to the true weight of the ox (Galton, 1907). In recent times, this idea has been popularly referred to as the “wisdom of the crowds” (Surowiecki, 2005). However, the effect of social cues in the form of collective decision on individual percept and the underlying neural mechanism remains largely unexplored (Klucharev et al., 2009; Izuma, 2013).

Neural expectation studies over the last decade have demonstrated that predictive cues typically lead to changes in early sensory processing (Carlsson et al., 2000; Kok et al., 2012a,b, 2013, 2014, 2016, 2017; Jiang et al., 2013; John-Saaltink et al., 2015; Todorovic et al., 2015; Sherman et al., 2016), but recent research has contradicted this claim (Bang and Rahnev, 2017; Rungratsameetaweemana et al., 2018). We sought to examine whether social information produces similar early top-down changes in the sensory cortex. We propose to manipulate the individual choice and decision confidence of humans performing a perceptual task by presenting visual cues that the subjects presume to be the collective opinion of other well-performing participants. The cues can be concurring, conflicting or neutral to the individual perceptual decision of the subjects. Using a novel statistical model, we studied the effect of the three types of cues on individual choice. We also analyzed the neural signals to explore the neural mediators producing the change in their individual choice upon being presented with social information. Finally, we performed a source reconstruction of the neural signals to elucidate the role played by specific spatio-temporal areas under the influence of cues. Specifically, we explored the following questions:

Can we manipulate individual perceptual decisions upon presenting potential social information cues when the cues differ from the individual choice? Does this reversal of opinion depend upon how confident the subject was in his/her choice without any influence from cues?

Can individual decision confidence be augmented when the cues concur with the individual choice?

Can we identify flip-floppers based on computational modeling of their behavioral data and corroborate using neural data?

Can we explore the neural mediators that contribute to the change in individual percept post-cue display?

Using a face/car discrimination task, we show that it is possible to manipulate individual choice post-presentation of cues in the guise of the decision of others. Although the cues were randomly generated and independent from the stimulus, it was possible to alter the individual percept, as subjects presumed the cues as concurring, conflicting, or neutral. Irrespective of the order in which they viewed the images with or without cues, most subjects were affected by the cues in a systematic manner. The distribution of the decision confidence under such a set up was found to be bimodal and skewed, with one mode guided by social information and the other influenced by the individual's own decision. The tendency to adhere to their own decision depends on the confidence level of the subject and is reflected in the skewness of the data distribution. Hence, using a Gaussian model to explore the data, which is the usual practice (Park et al., 2017), might not capture the complexities of data completely. We propose a novel model using a mixture of shifted gamma and negative gamma distributions that successfully captures the effect of social cues on individual choice. To the best of our knowledge, this is the first study using a mixture of variants of gamma distributions, which captures the bimodal nature as well as the skewness (whether high or low) of this kind of data. We compare our proposed model with the mixture of two Gaussian distributions and demonstrate the superiority of our model convincingly. Based on the behavioral model, it was possible to objectively identify subjects most prone to change their decisions upon being presented with the opinion of others. Subsequent multivariate pattern analysis (MVPA) of neural data substantiated the above finding. Neural analysis also elucidated the existence of a late component that seems to code the effect of this social information on individual perceptual decision. Source analysis of neural data revealed a role for the frontal cortex in coding perceptual decision using social information. Our analysis alludes to the role of the medial frontal cortex in coding information when conflicting social decisions are provided as cues.

## 2. Materials and Methods

### 2.1. Stimuli and Display

The data set consisted of 290 × 290 pixel 8-bit gray-scale images of 12 cars and 12 faces with an equal number of frontal views and side views. Face images were taken from the Max Planck Institute for Biological Cybernetics face database (Troje and Bülthoff, 1996). All stimuli were filtered to attain a common frequency power spectrum. Noise was generated by filtering white Gaussian noise (std of 3.53 cd/m^2^) by the average power spectrum. Noise was added to the base stimuli to generate a set of 250 images (125 face, 125 car). The contrast energy of all 250 images was matched at 0.3367 *deg*^2^. The participants were at a distance of 125 cm from a display with a mean luminance of 25 cd/m^2^. Images subtended a visual angle of 4.57°.

### 2.2. Participants and Experiment

**Figure 1:**
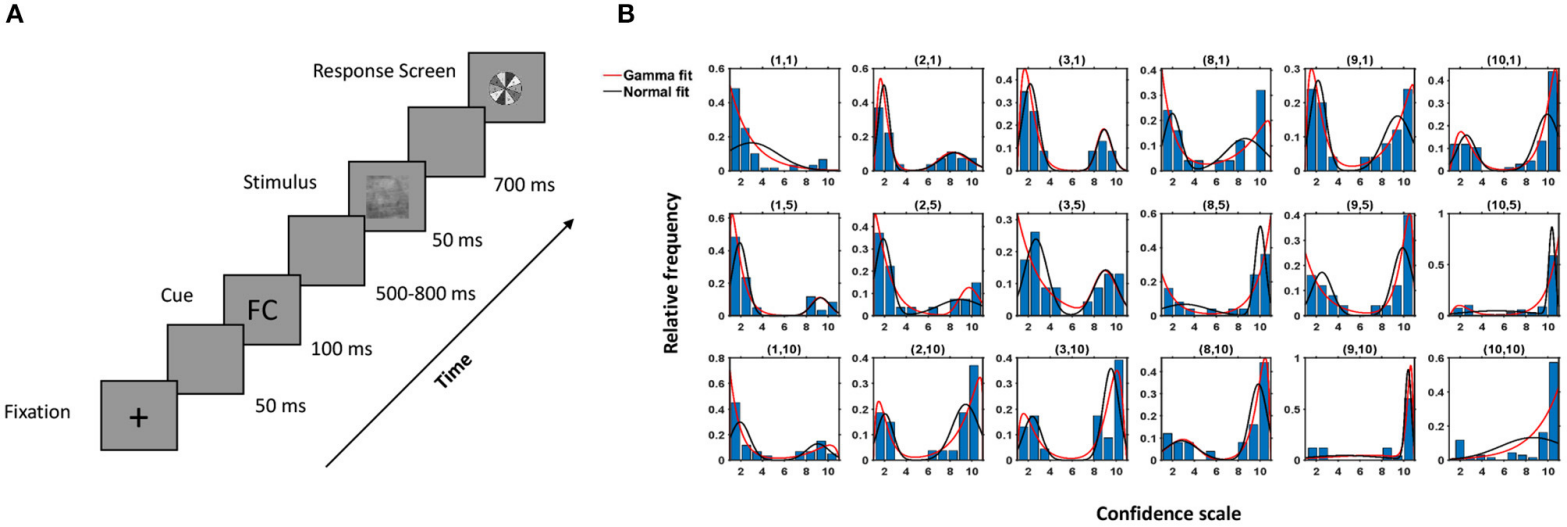
Experimental protocol and behavioral response. **(A)** Experimental Paradigm. **(B)** Histogram of the observed data and fitted density of the proposed model (red) and Gaussian mixture model (black) for a subject for different combinations of *k*_1_, *k*_2_ (denoted above each case, e.g., (1,10) implies subject data and fitted model for the images when individual choice was 1, denoting face with highest confidence, and cue shown was CC). Here, the x-axis denotes the confidence scale, and the y-axis denotes the relative frequencies of the subject's choices for a particular combination of *k*_1_, *k*_2_.

Twenty naïve participants (ages: 22–28, mean: 25.85, std: 2.39) participated in the study, which consisted of 1,000 trials split into 40 successive sessions. Three subjects were not considered in the analysis due to the high degree of noise present in the neural data. All participants had normal or corrected-to-normal vision and disclosed no history of neurological problems. The participants performed a face/car discrimination task and reported their decision using a 10-point confidence rating. Participants perceptually categorized briefly (50 ms) presented images of cars (C) and faces (F) embedded in filtered noise. The participants began by fixating on a central cross and clicking anywhere on the screen. After a delay of 50 ms, a cue was presented for 100 ms followed by a variable delay of 500–800 ms. The stimulus was presented for 50 ms followed by a delay of 700 ms, after which the response screen appeared. The participants reported their decision using the confidence rating, with a rating of 1 indicating complete confidence that the stimuli was a face and a rating of 10 indicating complete confidence that it was a car. The participants reported their confidence rating on a gray-scaled colorwheel in the response screen to avoid any motor bias (Figure 1A). There were four types of cues, FF, CC, FC, and CF, representing decisions of two independent well-performing participants who had previously completed the study. Cues were systematically manipulated such that an equal number of images (250 per condition) had FF cues, FC/CF cues, and CC cues. There were also an additional 250 images without cues. Thus, each participant saw one stimulus four times preceded by an FF cue, FC/CF cue, CC cue, and no cue in the course of the experiment in random order, and the responses were recorded. Participants were naïve to the purpose of the study and, in subsequent questionnaire after the study, failed to realize that the cues were not decision cues but were, in fact, synthetic cues generated randomly.

EEG activity was recorded using 64-channel active shielded electrodes mounted in an EEG cap following the international 10/20 system. EEG signals were recorded using two linked Nexus-32 bioamplifiers at a sampling rate of 512 Hz, band-pass filtered (0.01–40 Hz.) and then referenced using average referencing. Trials with ocular artifacts (blinks and eye movements) were detected using bipolar electro-occulograms (EOG) with amplitude exceeding ±100 mV or visual inspection and were not included in the analysis.

### 2.3. Behavioral Model

We propose a statistical model to explore the effect of the presented cues on perceptual decision making. In the experiment, for every face/car stimulus, subject responses corresponding to the three types of cues (FF, FC/CF, and CC) along with a response to the same stimuli with no cues were recorded. The response to the no-cue image was taken as the individual decision on the subject, *k*_1_ ∈ {1, 2, …10}, for that image. Further, we define a social cue variable *k*_2_ as

$$\begin{array}{l} k_{2}=\{\begin{array}{ll} 1 & \text{if cue shown was }‘\text{FF}’,\\ 5 & \text{if cue shown was }‘\text{FC}/\text{CF}’,\\ 10 & \text{if cue shown was }‘\text{CC}’. \end{array} \end{array}$$

All the images in which the individual decision of the subject was *k*_1_ were considered, and the distribution of the decisions on the same images under the influence of each type of cue was studied. Hence, the data comprised the decisions of a particular subject for every (*k*_1_,*k*_2_) pair. In most cases, the data distributions were bimodal in nature, having positive and/or negative skew, as seen in Figure 1B. Hence a two-component mixture model based on variants of the gamma distribution was proposed to explain the decisions taken by the subject under the influence of a cue. The data were made continuous by using jittering (addition of uniform random noise, Chanialidis, 2015) to provide flexibility in modeling.

Let **X**_*i*_(*k*_1_, *k*_2_) contain the decisions taken by the *i*th subject on all images, where his/her individual decision was *k*_1_ and the cue shown was *k*_2_. We consider the elements of **X**_*i*_(*k*_1_, *k*_2_) as i.i.d. observations from a distribution. To propose the statistical model depending on the choices of (*k*_1_, *k*_2_), we first introduce some terminology and notation. The probability densities of *shifted gamma* and *negative gamma* distributions are given, respectively, as

(1) $$\begin{array}{l} g\left(x\right)=\frac{\beta^{\alpha }}{\Gamma \left(\alpha \right)}{\left(x-1\right)}^{\alpha -1}e^{-\beta \left(x-1\right)},x\geq 1,\alpha \geq 1,\beta >0 \end{array}$$

(2) $$\begin{array}{l} ng\left(x\right)=\frac{\beta^{\alpha }}{\Gamma \left(\alpha \right)}{\left(L-x\right)}^{\alpha -1}e^{-\beta \left(L-x\right)},x\leq L,\alpha \geq 1,\beta >0, \end{array}$$

where α and β are the shape and scale parameters, respectively, and *L* is a known constant.

Based on Equations (1) and (2), the following models are proposed depending on the choices of (*k*_1_, *k*_2_). If *k*_1_ ∈ {1, 2, …, 5} and *k*_2_ ∈ {1, 5}, we take our model as

(3) $$\begin{array}{l} f\left(x\right)=p\;g_{\alpha_{1},\beta_{1}}\left(x\right)+(1-p)\;g_{\alpha_{2},\beta_{2}}\left(x\right), \end{array}$$

a mixture of two shifted gamma distributions. When *k*_1_ ∈ {6, 7…, 10} and *k*_2_ = 10, the proposed model is

(4) $$\begin{array}{l} f\left(x\right)=p\;ng_{\alpha_{1},\beta_{1}}\left(x\right)+(1-p)\;ng_{\alpha_{2},\beta_{2}}\left(x\right), \end{array}$$

a mixture of two negative gamma distributions. Finally if either *k*_1_ ∈ {1, 2, …, 5} and *k*_2_ = 10 or *k*_1_ ∈ {6, 7…, 10} and *k*_2_ ∈ {1, 5}, our suggested model is

(5) $$\begin{array}{l} f\left(x\right)=p\;g_{\alpha_{1},\beta_{1}}\left(x\right)+(1-p)\;ng_{\alpha_{2},\beta_{2}}\left(x\right), \end{array}$$

a mixture of a shifted gamma and a negative gamma distribution, where 0 ≤ *p* ≤ 1 is the mixing parameter.

#### 2.3.1. Parameter Space of the Model

We have taken the restricted parameter space for the shape parameter (α) in both the distributions (Equations 1 and 2) so that the modes of the distributions are defined and are either more than or equal to 1 (for the shifted gamma case) or less than or equal to *L* (for the negative gamma case). In our case, we consider *L* to be 11. In particular, for both shifted-gamma and negative-gamma distributions,

- the shape parameter α ∈ [1, ∞) and
- the scale parameter β ∈ (0, ∞).

#### 2.3.2. Estimation of the Model Parameters

Next, for the purposes of estimation of the parameters of our proposed model and further inference, only those data are considered that have more than 10 observations. Note that the parameter estimates depend on *i* as well as (*k*_1_, *k*_2_); that is to say, for every individual *i*, the parameter estimates may vary for different choices of (*k*_1_, *k*_2_). Similarly, for a given (*k*_1_, *k*_2_), parameter estimates of the proposed model may vary from individual to individual. We estimate the model parameters by a maximum likelihood estimation procedure (Casella and Berger, 2002). Since the proposed models are mixture densities, to calculate the maximum likelihood estimates (MLE) we invoke the EM algorithm technique (Casella and Berger, 2002). However, since closed-form solutions for estimates of shape parameters do not exist, we apply the Newton Raphson numerical technique (Atkinson, 1978) within each M-step of the EM algorithm (see Supplementary Information for detailed calculation).

#### 2.3.3. Goodness of Fit

To understand how well our model fits the observed data, the Kolmogorov-Smirnov (KS) test statistic (Gibbons and Chakraborti, 2011), based on the maximum absolute differences between the hypothesized cumulative distribution function (cdf) and empirical cumulative distribution function (ecdf), was used. For each subject *i*, there were *N*_*i*_ models to be tested simultaneously, and the case of multiple testing therefore arose. To control the family-wise error rate arising due to multiple hypothesis tests per subject, we used the Holm-Bonferroni method (Westfall et al., 1993) with a family-wise error rate (FWER) of 0.05.

#### 2.3.4. Model Prediction

We use a 10-fold cross-validation procedure to study the predictive performance of the proposed model. Since our data were bimodal in nature, it would not have been meaningful to judge this performance on the basis of a single predictive interval. To address this issue, we applied the following concept of a highest probability density region (HPDR) (Hyndman, 1996), which broadly computes the smallest region that contains most of the probability.

***Definition***: Let *f*(*x*) be the probability density function of a random variable *X*. The 100(1 − α)% HPDR is then defined as the subset *R*(*f*_α_) of real numbers, ℝ, such that

$$\begin{array}{l} R\left(f_{\alpha }\right)=\left\{x:f\left(x\right)\geq f_{\alpha }\right\}, \end{array}$$

where *f*_α_ is the largest constant with *P*(*X* ∈ *R*(*f*_α_)) ≥ 1 − α.

In each fold, the model was trained on the training set, and the 95% HPDR was computed. It was checked whether the validation set fell within the estimated HPDR, and the process was repeated for each cross-validation fold.

#### 2.3.5. Model Comparison

We compared the performance of our proposed model with the two-component Gaussian mixture model using a likelihood ratio test (Casella and Berger, 2002). Data were divided into 10 test sets using 10-fold cross-validation and, for each set, the likelihood was estimated with each of the two models. Finally, the medians of the likelihood ratios across the folds were computed for each of the models for the purpose of comparison.

### 2.4. Behavioral Data Processing

Guided by the proposed model, the behavior of the individuals were analyzed based on the following measures.

#### 2.4.1. Distance Metric Computation Using the Model

To quantify the overall shift in decisions from the subjects' individual choice, the following distance was used

(6) $$\begin{array}{l} \begin{array}{l} D_{i}(k_{1},k_{2})=\{\begin{array}{ll} \sqrt{x_{i}^{\prime }x_{i}} & \text{ if }k_{1}=k_{2},\\ \sqrt{x_{i}^{\prime }\Sigma^{-1}x_{i}} & \text{ otherwise,} \end{array} \end{array} \end{array}$$

where $x_{i}=(k_{1}-m_{1}(i),k_{1}-m_{2}(i){)}^{\prime }$, **m_1_** and **m_2_** being the vectors containing the two modes of the *N*_(*k*__1_, *k*_2_) subjects and *i* = 1, 2, …, *N*_(_*k*__1_, *k*_2_)_. Here, *N*_(_*k*__1_, *k*_2_)_ denotes the number of subjects available corresponding to (*k*_1_, *k*_2_), and Σ is the estimated variance covariance matrix of estimates of the modes for a particular choice of (*k*_1_, *k*_2_), given by

$$\begin{array}{l} \Sigma =\left[\begin{array}{cc} \text{Var}(m_{1}) & \text{Cov}(m_{1},m_{2})\\ \text{Cov}(m_{1},m_{2}) & \text{Var}(m_{2}) \end{array}\right]. \end{array}$$

#### 2.4.2. Social Bias Score

Using the cumulative distribution functions of shifted-gamma and negative-gamma distributions (as calculated in Supplementary Information) and Equations (3)–(5), the proportion of decisions between *k*_1_ and *k*_2_ in the presence of social cues was estimated. The average proportion of decisions (*p*_*i*_) per subject across the (*k*_1_, *k*_2_) pairs, which are reported in Tables S5–S8, was considered. We ranked the subjects based on social bias score, defined as

$$\begin{array}{l} W_{i}=\frac{p_{i}-0.5}{\sigma /\sqrt{n}}, \end{array}$$

for *i* ∈ {1, 2, …, 17}\{2, 3}, with σ denoting the sample standard deviation of the proportions *p*_*i*_. Only those subjects were considered for further analysis whose *W*_*i*_ exceeded 1.96, indicating that the corresponding proportions are significantly more than accounted for by chance.

### 2.5. Neural Data Processing

The preprocessed EEG signals were time-locked to stimulus onset and included a 200 ms pre-stimulus baseline and 500 ms post-stimulus interval.

#### 2.5.1. Multivariate Pattern Analysis of EEG

Univariate EEG analysis had traditionally been used to explore the relationship between behavioral performance and neural activity in specific cognitive tasks. However, the univariate analysis techniques fail to fully utilize the spatio-temporal nature of multivariate neural data. Multivariate pattern analysis techniques provide a way to integrate the spatial and temporal information present in the data by fusing the neural information into a single decision variable that can be used in single-trial analysis. A comparison between univariate and multivariate analyses using a similar cognitive task has been shown in Das et al. (2010). Successful use of MVPA has been demonstrated in numerous studies using EEG and fMRI (Haynes and Rees, 2005; Kamitani and Tong, 2005; Philiastides et al., 2006). In the current study, MVPA was used to extract meaningful information from the multi-dimensional EEG data. Since the neural data is high dimensional and suffers from the small sample size problem (Das and Nenadic, 2009), a recently proposed principal component analysis (PCA)-based non-linear feature extraction technique–“Classwise Principal Component Analysis” (CPCA) (Das and Nenadic, 2009)–is used. CPCA has been used previously to efficiently reduce the dimensionality of the EEG signals and extract informative features (Das et al., 2009, 2010; Do et al., 2011, 2013; Wang et al., 2012; King et al., 2013). The main goal of CPCA is to identify and discard non-informative subspace in data by applying principal component-based analysis to each class. The classification is then carried out in the residual space, in which small sample size conditions and the curse of dimensionality no longer hold. A Linear Bayesian Classifier was then used for computing the choice probability for single-trial EEG data for each subject. Pattern analysis was performed using 10-fold cross-validation. The original data were partitioned into 10 equally sized subsamples. Of the 10 subsamples, a single subsample was retained as the test data, and the remaining nine subsamples were used in training the classifier. The performance of the classifier is captured by the receiver operating characteristics (ROC) curve, which plots the true positive rate vs. false positive rate at different classification thresholds. The area beneath this ROC curve (AUC) is often used as a measure to determine the overall accuracy of the classifier (Duda et al., 2012). We utilize the well-known approach of calculating the area under the ROC by finding the Mann Whitney U-statistic for the two-sample problem (Mason and Graham, 2002). All classification analyses were carried out for individual participants, and the average AUC performance was reported in the results.

#### 2.5.2. Source Reconstruction

To identify underlying neuronal sources responsible for generating differences in the ERPs corresponding to the face and car trials under the influence of cues, source reconstruction was performed using sLORETA software (Pascual-Marqui, 2002, http://www.uzh.ch/keyinst/loreta). sLORETA (standardized low-resolution brain electromagnetic tomography) is based on standardization of the minimum norm inverse solution, which considers the variation of actual sources and the variation due to noisy measurement (if any) as well (Pascual-Marqui, 2002). As a result, it does not have any localization bias, even in the presence of measurement and biological noise. The head model for the inverse solution uses the electric potential lead field calculated using the boundary element method (Fuchs et al., 2002) on the MNI152 template (Mazziotta et al., 2001). The cortical gray matter is partitioned into 6,239 voxels at 5-mm spatial resolution. sLORETA images represent the standardized electric activity at each voxel in Montreal Neurological Institute (MNI) space as the exact magnitude of the estimated current density. Anatomical labels are reported using an appropriate correction from MNI to Talairach space (Talairach and Tournoux, 1988) using Talairach Daemon (Lancaster et al., 2000). For further details on sLORETA, refer to http://www.uzh.ch/keyinst/NewLORETA/Methods/MethodsSloreta. The source activity was estimated from the face-car difference wave post-stimulus onset.

#### 2.5.3. Statistical Analysis of Sources

Differences in the distribution of the sources between concurring and conflicting trials were calculated using statistical non-parametric mapping (SnPM) (Nichols and Holmes, 2002). This method relies on the randomization of the absolute maximum statistic over all channels. The randomization provides an estimator for the empirical distribution under the null hypothesis (“no difference between the sources of concurring and conflicting trials”). The advantage of this method is that it does not depend on any distributional form, in particular Gaussianity, and simultaneously takes care of multiple comparisons. A total of 5,000 random samples were generated while implementing the SnPM technique. Differences between the two conditions (concurring and conflicting) were assessed at the global level, and the brain areas showing the largest differences have been reported.

## 3. Results

### 3.1. Behavioral Results

The decisions taken by the subjects under the influence of a cue were modeled as a two-component mixture model based on the shifted-gamma and negative-gamma distributions (see Equations 3–5). To verify that the proposed model fits the observed behavior data well, the Kolmogorov-Smirnov (KS) test (Gibbons and Chakraborti, 2011) was used. The proposed model captured the data correctly in most cases (see Table S1). Figure 1B depicts histograms of the decisions corresponding to all (*k*_1_, *k*_2_) pairings and the fitted density of our model for one subject. Table S1 contains the *p*-values corresponding to the cases where the model was rejected. In over 96% of the cases, the hypothesized model was accepted, thus proving efficacy of the model.

**Figure 2:**
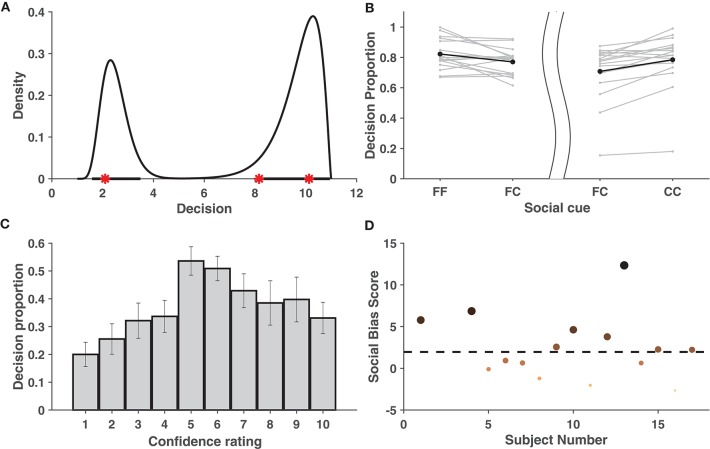
Behavioral Data Analysis. **(A)** Estimated probability density function based on the training data set shown for one subject when *k*_1_ = 3 and *k*_2_ = 10. Bold lines on x-axis represent the 95% HPDR, and red stars represent the test observations for a subject. The test observations fall within the HPDR. **(B)** Increase in average proportions of decisions around the individual decision when viewing concurring cues vs. viewing neutral cues. The left part of the figure considers cases when the individual decision was a face, while the right part considers cases when it was a car. The bold dots depict the average across the individuals. **(C)** Mean proportion of decisions toward conflicting cues across individuals. Figure shows that crossover happens for all cases of individual confidence and is most prominent when individual decision confidence is low (5,6). Error bars denote ± SD. **(D)** Social bias ranking of subjects, indicating their tendency to be influenced by the cue shown. Larger and darker dots indicate subjects that are more socially influenced. The dotted line parallel to the x-axis depicts the significance level.

To measure the predictive performance of the proposed model and prevent possible over-fitting, after computing the highest probability density region (HPDR) of the fitted model based on the training data, it was checked whether the test data fell within the calculated HPDR. Table S2 showing mean prediction error rates across subjects, demonstrates that the cross-validation error rate never exceeded 5% for any fold, thus validating the excellent performance of the model in terms of prediction and nullifying the chance of over-fitting. Figure 2A shows a fitted density function and the corresponding HPDR calculated from the training data of a particular validation fold of one subject. The test data, as seen from the figure, falls convincingly inside the indicated HPDR.

Gaussian distribution has been previously used to model behavioral data successfully (Park et al., 2017). Hence, the proposed model was compared with the mixture of two component Gaussian distributions. The median of the likelihood ratios across subjects for a given (*k*_1_, *k*_2_) in all but two cases (out of 30) clearly indicates that the proposed model outperformed the Gaussian mixture model in terms of explaining the data (refer to Table S3).

#### 3.1.1. Effect of Cues on Individual Choice

The effect of cues on individual decision was studied using a distance metric between *k*_1_ and the estimated modes of the fitted model (see Equation 6). Using a bootstrap resampling technique on mean distance per (*k*_1_, *k*_2_) pair, it can be observed that post-cue, there was a significant shift in ratings when decisions from all subjects were pooled together (Table S4). Furthermore, to check whether this was also true for individual decisions, an additional analysis was carried out. If the proposed model predicted a mode in the direction of the social cue, the proportion of decisions between *k*_1_ and *k*_2_ was calculated by integrating the estimated density within the said interval. A significant proportion of decisions, as assessed by our model, was observed to lie between *k*_1_ and *k*_2_ (refer to Tables S5–S8), clearly suggesting that, in general, subjects tend to be influenced by the social choice, irrespective of whether it conforms to his/her individual bias.

#### 3.1.2. Effect of Concurring Cues

In order to check whether the decision confidence increased when the subject was given a cue concurring with his/her own judgment, the area under the fitted density given the concurring cue (“FF,” “CC”) was compared with that of a neutral cue (“FC”/“CF”) (see Tables S9, S10). These areas were assumed to be indicative of the proportion of decisions of the subjects around the individual decision. As compared to the neutral cue, for most of the subjects, the average proportion of decisions in the region [1,6] was greater when individual choice was a face and the social cue was also a face. Similarly, this proportion in the region [7,11] was greater when the individual and social choices were both a car. Thus, it can be concluded (refer to Figure 2B) that the decision confidence of most subjects increased when provided with concurring social information (FF/CC).

#### 3.1.3. Effect of Conflicting Cues

Further analysis was carried out to check whether there was a significant reversal in the decisions when the subject faced a cue contradictory to his/her individual decision. We say that there is a *cross-over* if there exists a mode on the opposite side of the decision boundary. Cross-over under the influence of concurring cues was found to be insignificant (in terms of area) compared to with conflicting cues (see Table S14) and was hence ignored. For every *k*_1_, it was examined whether cross-over exists given a mismatch between social cue and the individual choice. Using bootstrapping, it was shown that the proportion of cross-over was significant among the individuals. This is evident from the approximate achieved significance level (ASL) (Efron and Tibshirani, 1994) contained in Table S11. Figure 2C distinctly reveals that the mean cross-over proportion increased with a decrease in individual confidence, implying that, in general, subjects tend to be influenced more by contradictory cues on images where their individual confidence was low. Refer to Tables S12, S13 for a detailed list of the cross-over proportions per subject.

#### 3.1.4. Cue-Based Ranking of Subjects

Individuals differ in the manner in which social information influences their perceptual decision. Using the proposed behavioral model, it is possible to rank the subjects based on the level of influence social information had on their percept. Figure 2D shows the ranking of subjects based on a measure, called social bias score, that captures their tendency to be influenced by social information. Based on the analysis, eight^1^ subjects were selected as those most affected by cues and are referred as *chosen subjects* in the EEG analysis.

### 3.2. Neural Results

#### 3.2.1. ERP Analysis

**Figure 3:**
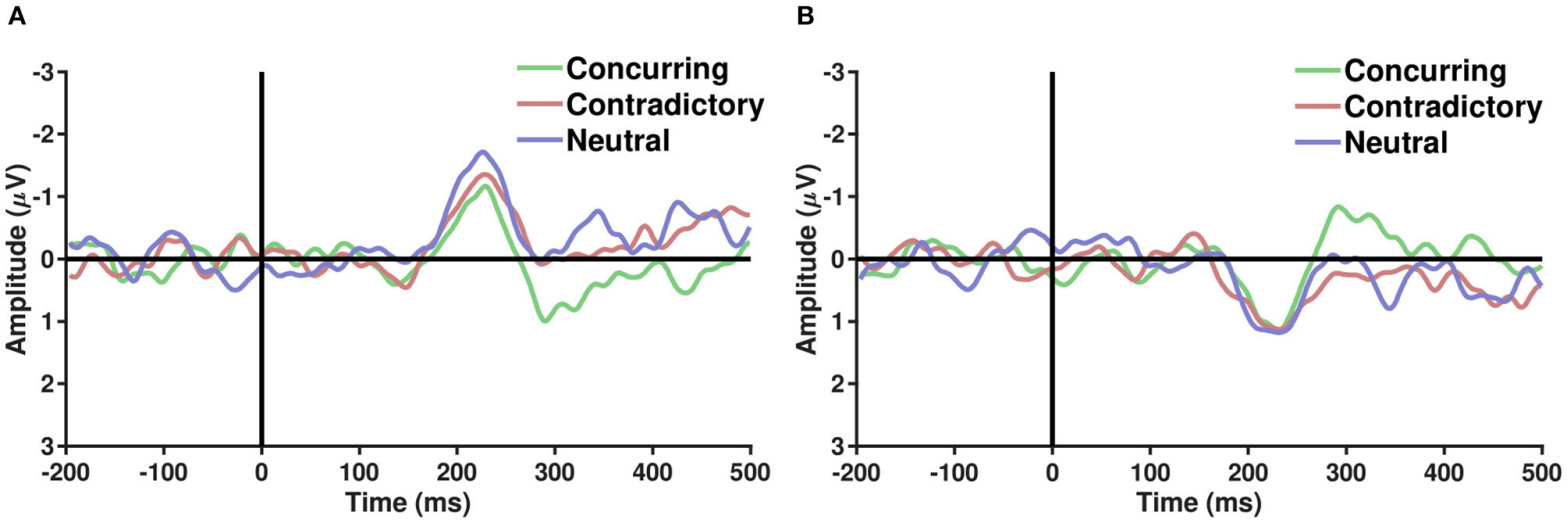
**(A,B)** Show the grand average of difference ERPs (Face − Car) over parieto occiptial and fronto central electrodes, respectively, across the three types of conditions—Concurring, Conflicting, and Neutral. A sharp peak in the difference waveforms is observed post-200 ms across all conditions. Difference between conflicting and concurring cues seems more prominent around 250–300 ms.

ERP analysis was performed on average referenced and baseline-subtracted EEG signals for each condition. Epochs of a particular channel were marked noisy if their respective absolute differences from the median exceeded five times the interquartile range. Such noisy epochs were not considered for further ERP analysis. It is well-known that parieto-occipital electrodes show differential activity when perceiving faces and cars (Rossion et al., 2003). Several studies have hypothesized the role of the frontal cortex in choice manipulation under the influence of social information (Mason and Graham, 2002; Berns et al., 2010; Klucharev et al., 2011; Izuma and Adolphs, 2013). To explore the effect of the decision of others on face/car percepts, ERP analysis was carried out with parieto-occipital and fronto-central electrodes separately. To elucidate whether different types of comments induce different neural processing mechanisms, the grand average difference waves were plotted (refer to Figure 3) for correctly guessed face and car trials. A difference in face and car ERPs was visible across both fronto-central and parieto-occipital electrodes around 200 ms post-stimulus onset, closely following the N170 (Bentin et al., 1996) component known to be enhanced more in face than non-face ERPs. The difference between concurring and conflicting conditions, however, seemed more prominent around 250–300 ms post-stimulus condition in both parieto occipital and fronto central electrodes. Further analysis was carried out using single-trial multivariate analysis.

#### 3.2.2. Single-Trial Multivariate Analysis

A pattern classifier was used to analyze single-trial EEG signals corresponding to the different types of cues. To quantify the predictive accuracy of the classifier, the posterior probabilities obtained from 10-fold cross-validation were used to calculate the area under the ROC curve (AUC). The AUCs were averaged across the subjects.

**Figure 4:**
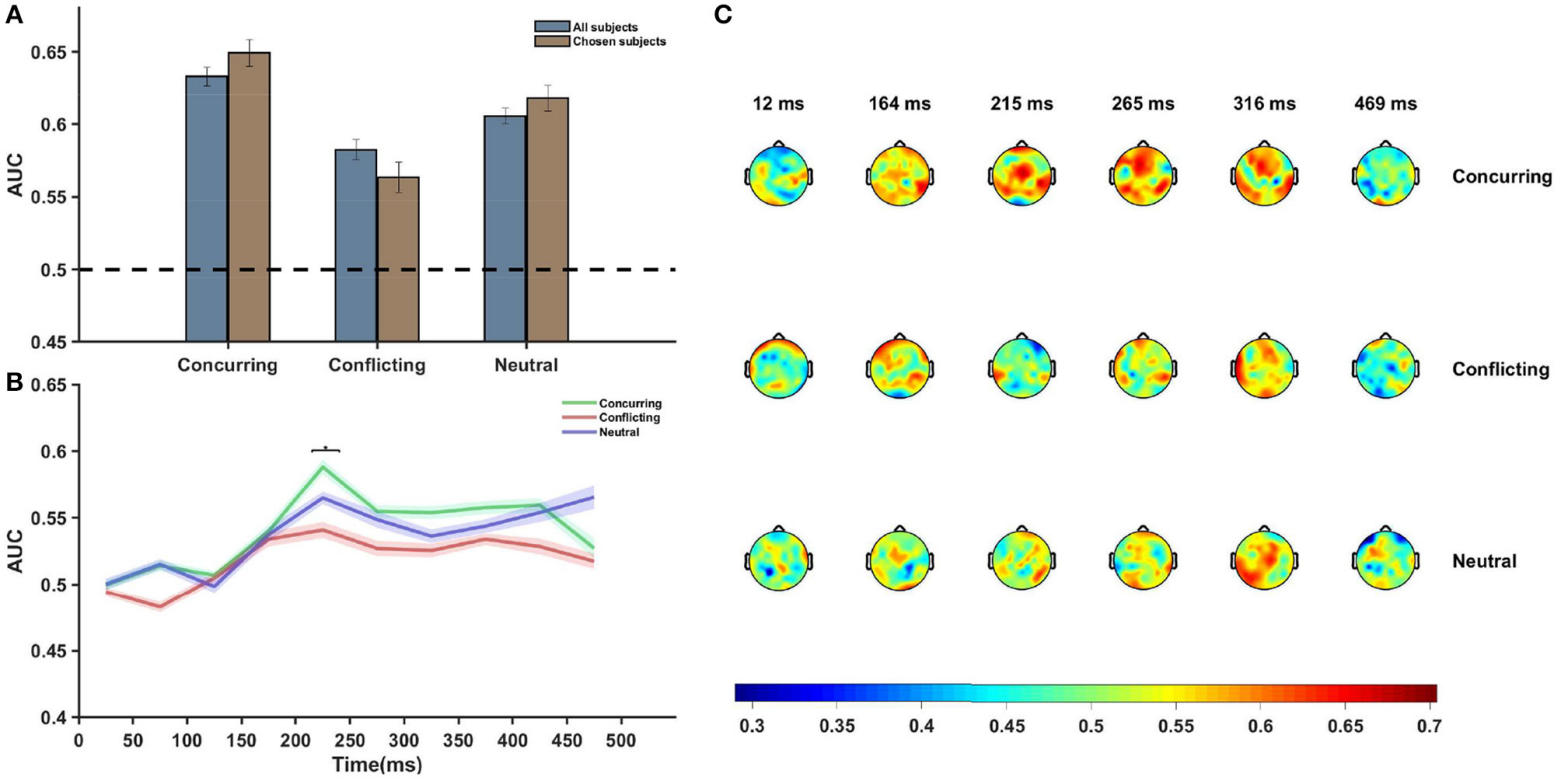
Neural data analysis. **(A)** Figure shows average AUC predicting choice probability with single-trial EEG analysis and using multivariate pattern analysis. Average AUC increases under the influence of concurring cues and decreases under the influence of conflicting cues as compared to that for neutral cues in all subjects. The effect is more prominent in case of the chosen subjects. Error Bars indicate ± SEM. **(B)** Plot of average AUC across all subjects at different time points. The increase in AUC is most pronounced in the 200–300 ms post-stimulus interval. The difference between the AUCs of concurring and conflicting trials is most significant (*p* = 0.054, FDR corrected) in the 200–250 ms window (marked using *). **(C)** Topoplot of one subject showing per electrode per time window single trial classification under different cue conditions. Average AUCs of the all channels for successive time windows are shown. There appears to be a significant involvement of the frontal and occipital electrodes 200–350 ms post-stimulus onset. Color bar depicts the value of AUC.

Multivariate analysis was performed using the entire post-stimulus dataset using all channels and all time points, and AUCs corresponding to the different conditions were plotted (Figure 4A). The classification accuracy appeared to be greater when the subject was provided with a cue that concurred with his/her individual guess than when he/she was provided with a conflicting cue (*p* = 0.0213, *df* = 14, *t* = 2.2314). An overall increase in difference was noted between the conditions (*p* = 0.0038, *df* = 7, *t* = 3.7147, corresponding to the null hypothesis of no difference in the classification rates between the two conditions) when an average over chosen subjects was considered (Figure 4A). The pattern analysis was executed separately using EEG data for all electrodes across different time windows, each having a length of 50 ms. AUCs corresponding to the late sensory period (200–450 ms after stimulus onset) were found to be significantly more than chance (*p*-value < 0.05, false discovery rate (FDR) corrected) for concurring trials.

Further analysis showed that the difference between AUCs of concurring and conflicting cues was statistically significant only in the time window 200–250 ms [*p*-value (without multiple correction) = 0.01, *t* = 2.585, *df* = 14, FDR corrected *p*-value = 0.054, multiple hypothesis test performed across time points where the classification rates corresponding to concurring trials are more than chance]. On performing similar time-window analysis on the chosen subjects, it was seen that the difference stood out as statistically significant [*p*-value (without multiple correction) = 2.40 × 10^−5^, *t* = 8.8377, *df* = 7, FDR corrected *p*-value << 0.05] in the 200–250 ms time window.

Figure 4B clearly depicts that around 200–250 ms after stimulus onset, there was a sharp increase in the AUC value and the peak was more pronounced for concurring cues. Notably, prominent activity in fronto-central and occipito-temporal electrodes in a similar time window was also observed during ERP analysis.

Additional classifier analysis was carried out using data for each electrode separately for each of the time windows (Figure 4C), and the plot of scalp topography on the basis of the classifier performances (see Figure 4C) for individual electrodes seems to be consistent with the temporal findings (Figure 4B). Around 200–300 ms post-stimulus onset, we observe increased classification accuracy in the parieto-occipital regions and fronto-central regions across all conditions (concurring, conflicting, and neutral). In these regions, the magnitude of the AUCs were greater in case of concurring trials than in conflicting and neutral trials (see Figure 4C). The classifier results demonstrate that social decisions have an effect on individual perceptual decision and that it is most prominent around 200–300 ms post stimulus onset.

#### 3.2.3. Source Reconstruction Results

**Figure 5:**
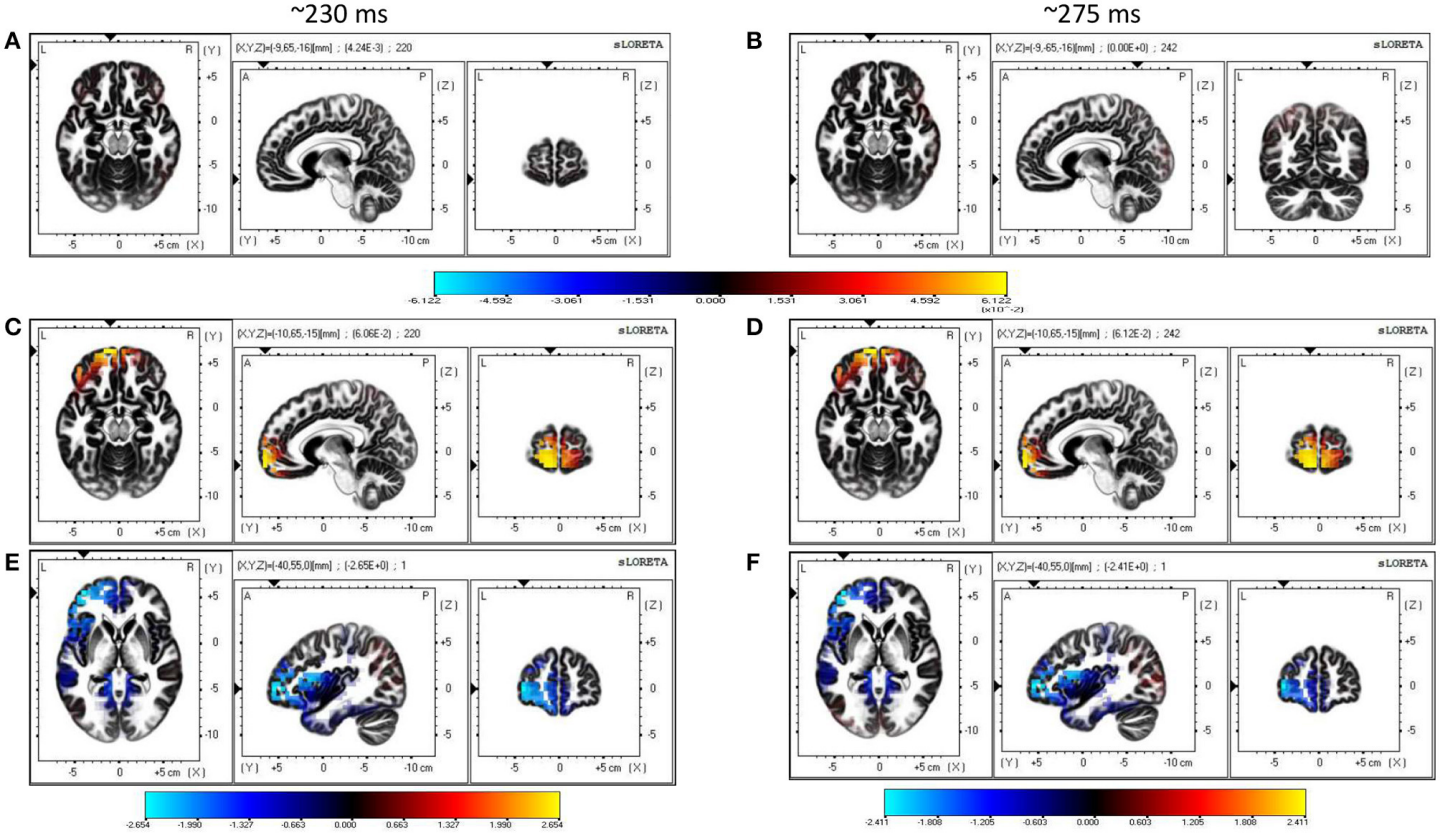
Source Reconstruction. **(A,B)** Show sources estimated at 230 and 275 ms using sLORETA software for trials with concurring cues. **(C,D)** Show sources estimated at 230 and 275 ms using sLORETA software for trials with conflicting cues. The color bar depicts the squared magnitude of the current density [μ*A*^2^/(*mm*^4^.*Hz*)]. **(E,F)** Are maps of non-parametric statistics comparing concurring and conflicting trials during the 200–250 and 250–300 ms time windows. Non-parametric analysis was performed using one-tailed F-ratio test (concurring < conflicting) on paired data. Color bar represents value of log F-ratio for each voxel.

Single-trial multivariate data analysis and ERP analysis revealed prominent discriminatory activity 200 ms post-stimulus onset. Source estimates identified more frontal activity under the influence of conflicting cues than with concurring cues (refer to Figure 5). Frontal sources seem to be primarily responsible for generating differences in the ERP waveforms of face and car trials across the whole neural timeline for conflicting trials, while a prominent fronto-parietal interplay was noticed in case of concurring and neutral trials. Particularly, the medial frontal gyrus seems to have contributed significantly in the presence of conflicting cues, in line with previous studies that also highlight the role of the medial frontal cortex during social conformity and cognitive dissonance (Klucharev et al., 2009; Berns et al., 2010; Izuma and Adolphs, 2013). The neural sources of the difference in the current density power between the concurring and conflicting conditions were analyzed using sLORETA with a one-tailed F-ratio test (concurring < conflicting) on paired data separately for the 200–250 and 250–300 ms time windows. Based on the results of the exceedance proportion test (Friston et al., 1990, 1991) which showed a threshold of 2.38 for a *p*-value of 0.058 for the 200–250 ms window and a threshold of −2.169 for a *p*-value of 0.059 for the 250–300 ms window, differences were localized mostly to the frontal areas (refer to Tables S16, S17 for the complete list). We found the maximal differences in the medial frontal gyrus (BA 10, MNI coordinates: *x* = 40, *y* = 55, *z* = 0) in both the cases (refer to Figures 5E,F and Tables S16, S17).

#### 3.2.4. Neural Analysis of Cue Data

**Figure 6:**
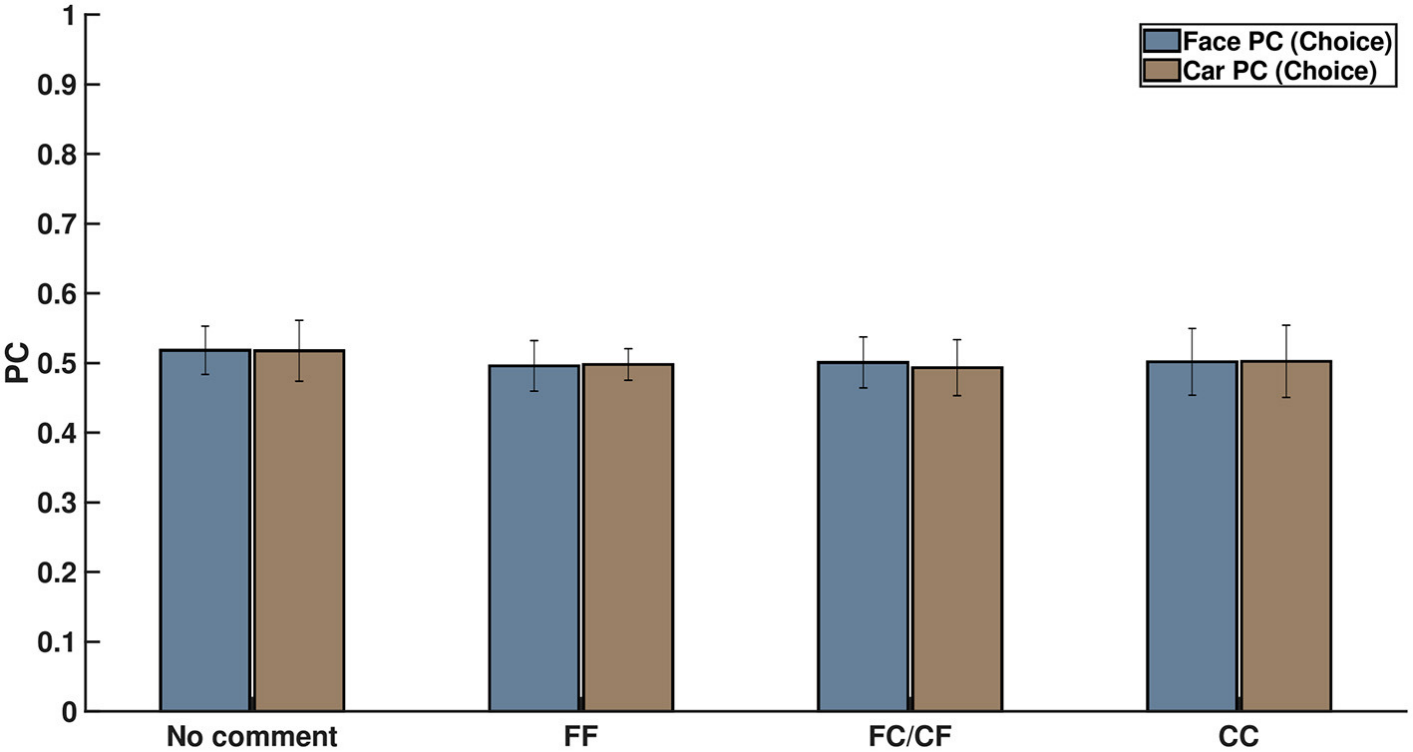
Percentage of correctly classified face and car decisions for the four kinds of comments shown on screen on the basis of their neural signals after cue exposure. This clearly shows that subject choice did not arise from cue-related expectation bias.

We did an additional analysis based on the neural signals when the cue was displayed. We extracted the EEG signals locked to the cue onset. The 500-ms post-cue onset data were used to perform multivariate pattern analysis to explore the effects of expectation on early sensory processing. If the participants' responses were driven by the cues, then we would expect a higher classification rate for images selected as faces post-cue onset when preceded by an “FF” cue and vice versa for “CC” cues. However, pattern analysis of cue-data revealed no such trends (refer to Figure 6) and resulted in chance performance for all conditions (*p* > 0.05). Two-way ANOVA was performed to find the statistically significant difference between the four different cue conditions, taking into account face and car trials separately, along with interactions. The differences were all insignificant (see Table S15), pointing to the fact that there was no significant difference in the classification accuracy across all the cue conditions, including the condition where no cue was shown. It is interesting to note that similar chance performance was also observed in pre-stimulus and early post-stimulus (<200 ms) neural classification. Thus, based on the cue analysis, it seems unlikely that the participants' decision was influenced by cue-based expectation bias in the post-cue onset and early visual processing stage following the stimulus display.

## 4. Discussion

How social decision affects individual decision-making has been explored in social psychology since the 1940s, starting with the research on social conformity by Solomon Asch (Asch and Guetzkow, 1951; Asch, 1955; Tajfel, 1982). With the advent of social media, there has been a renewed interest in social cues influencing our decisions (Jansen et al., 2009; Bond et al., 2012; Gonçalves and Perra, 2015; Jayles et al., 2017). In the current study, how people respond to social information when performing a perceptual decision-making task was explored systematically. The neural mechanism of the decision-making process was studied while the subjects used cues in the form of the decision of two other well-performing subjects to perceive noisy images of faces and cars. Although the cues shown to the subject were non-informative, with an equal number of FF, neutral, and CC cues per stimulus displayed in a random order, they were found to be successful in manipulating percept. Most of the studies on social influence require participants to make a decision with and without social cues sequentially, but we demonstrate that, irrespective of the order in which the stimulus/cue was presented, cues always have a similar effect on individual decision-making. We conclude that the perceptual decision of the subject under the influence of the cue depends on two factors—his/her individual perception of the image, as reflected in his/her confidence ratings on the same images without any cue, and the social information presented to him/her. It is observed that the distribution of confidence ratings under the influence of a cue is bimodal in nature, with one mode corresponding to individual decision and other to social cue (Figure 1B), with a significant proportion in the direction of the cue. We can thus safely infer that although there was a general tendency to adhere to one's individual decision, subjects' decision confidence could be altered by social influence. This shift in decision confidence varied between the subjects, as reported in previous studies (Jayles et al., 2017). Using the proposed computational model, the heterogeneity of the influence of cues on the subjects' decision was quantified successfully. The subjects were ranked based on the influence the cues elicited, and the findings used in subsequent neural analysis produced encouraging results.

Although social influence on perceptual decisions remains a highly researched topic, the neural mediators of the manipulation of perceptual decisions by social influence remain largely unexplored (Mason and Graham, 2002; Berns et al., 2010; Klucharev et al., 2011; Izuma and Adolphs, 2013). The difference in performance under the influence of concurring and conflicting cues is most prominent in the 200–300 ms interval. Similar differences between conflict and no-conflict trials have been reported in recent papers (Shestakova et al., 2012; Zubarev et al., 2017). This time interval can potentially reflect an interaction between the social cues provided and the sensory information. It is interesting to note that the time window corresponds with the timing of feedback-related negativity (FRN) (Holroyd and Coles, 2002) and task difficulty (Philiastides et al., 2006). The mean AUC value peaks around 200–300 ms in trials with concurring cues. This implies that the classifier could identify the class-specific discriminatory activity and predict the participants' decision more accurately when the cue received matched with his/her individual perception. This corroborates our claim that the subjects were more sure about their decisions when the stimulus was preceded by a concurring cue. The effect is more well-defined in case of car trials, probably arising out of heavier mental load for car images than faces. Humans are adept at face perception (Leopold and Rhodes, 2010), and the stimuli displayed had uniform noise for both faces and cars, thereby making the car-detection task comparatively difficult. Figure 2B shows this effect for concurring cues, where the increase in decision confidence was more prominent for CC cues than for FF cues. A similar trend is noticed for conflicting cues (Figure 2C), where significant reversal of decision in the direction of the social information was noticed and the proportion of crossover was more for trials originally detected as cars. Almost all the existing neuroimaging studies using social cues suggest the role of the posterior medial frontal cortex (pMFC) and, to some extent, the ventral striatum (Klucharev et al., 2009; Berns et al., 2010; Izuma and Adolphs, 2013) in social conformity, but the neural mechanism remains poorly understood. Current research shows that activation in the pMFC is modulated by the difference between individual choice and group preference. The role of the pMFC in social conformity is further strengthened by a TMS study (Klucharev et al., 2011) where participants showed reduced social conformity when the pMFC was disrupted. One plausible interpretation of the involvement of the pFMC could be that conforming to social opinions triggers similar circuitry as does reinforcement learning (Klucharev et al., 2009). Neural activity in the pMFC might mirror activity similar to a prediction error signal, which can then subsequently be used to modify or strengthen the perceptual decision. In the current study, source analysis of ERP signals using conflicting cues also shows activity in the medial frontal cortex (MFC), starting around 200 ms post-stimulus onset. Neural signals following conflicting cues displayed comparatively greater frontal activity than concurring cues (Figure 5), possibly suggesting greater top-down processing of information when cues mismatch perceptual choice. It is particularly interesting to note that the MFC is active in the time interval immediately following the well-established N170 component, which is known to account for the difference between faces and cars (Daniel and Bentin, 2012). Possibly, the mismatch between the top-down expectation produced by the cue and the bottom-up sensory information triggered activity in the MFC, which has been reported to play a role in social conformity (Klucharev et al., 2009; Izuma, 2013). The medial frontal cortex perhaps generates a signal that encodes the difference between individual percept based on the stimulus and the group decision given by the cues. The absence of frontal activity in concurrent cues in the same time interval further supports our claim. The strength of MFC activity has been shown to regulate the level of subsequent adjustment of individual choice (Berns et al., 2010). Hence the MFC activation was more pronounced for chosen subjects. Our results seem to suggest that, irrespective of stimulus order, neural circuitry similar to existing social conformity studies was active in making perceptual decisions under the influence of social cues.

There has been extensive research on face and object perception in the last few decades that has revealed significant involvement of various occipito-parietal regions in the early stages of visual processing (<200 ms) (Rossion et al., 2003). Additionally, there a significant body of work finding that stimulus expectation leads to changes in early sensory processing (Carlsson et al., 2000; Kok et al., 2012a,b, 2013, 2014, 2016, 2017; Jiang et al., 2013; John-Saaltink et al., 2015; Todorovic et al., 2015; Sherman et al., 2016). It has been demonstrated in numerous studies that expectation about stimulus in the form of predicting cues leads to a stimulus bias. Top-down expectation effects can be seen in the form of improvement in stimulus representation (Kok et al., 2012a), generation of a stimulus template in striate and extrastriate regions (Puri et al., 2009; Kok et al., 2014), and even reduction in amplitude in neural signals leading to “expectation suppression” effect (Todorovic and de Lange, 2012). On the whole, top-down expectations in the form of predictive cues have been shown to bias neural activity in the pre-stimulus and early sensory processing stage, thereby orienting the bottom-up sensory information toward one perceptual decision. On the other hand, recent studies have questioned the role of neural expectation in the sensory cortex (Bang and Rahnev, 2017; Rungratsameetaweemana et al., 2018). In our study, however, probing into the neural time series unveiled no significant differences in perception under the influence of different social cues during early stages. We systematically analyzed the effect of social decision and found no significant effect of the cues before stimulus onset, post-cue onset, and immediately following stimulus onset. We extracted the neural data locked to cue presentation and used a multivariate pattern classifier on the cue data alone to show that the cue data were not indicative of any early top-down expectation based effect on the stimuli (see Figure 6). Our results seem to suggest, unlike studies involving predictive cues (Summerfield and De Lange, 2014), that expectation by virtue of social influence does not affect early sensory processing. It is worthwhile to note here that our cues were essentially social decisions of others instead of cues predictive about the stimulus itself (Summerfield and Koechlin, 2008; Summerfield and De Lange, 2014), which could possibly explain the lack of top-down expectation signals seen in the early sensory cortex in previous studies (Summerfield and De Lange, 2014). Our results seem to suggest the role of downstream processing in using the social information from the cue provided, similar to the concepts of Bayesian Decision Theory (Maloney and Mamassian, 2009) and Signal Detection Theory (Green and Swets, 1988; Macmillan and Creelman, 2004).

Overall, we conclude that perceptual decision and confidence are influenced by social information and that it is possible to compute the extent of influence using statistical modeling. Neural data analysis alludes to a role for the medial frontal cortex in perceptual decision under social influence. We found no expectation-related bias in early sensory processing using social information cues. Future studies could possibly focus on experiments using actual social groups to validate the neural results found in the current research.

## Data Availability Statement

The datasets generated for this study are available on request to the corresponding author.

## Ethics Statement

This study was carried out in accordance with the recommendations of Institute Ethics Committee at Indian Institute of Science Education and Research Kolkata, India with written informed consent from all subjects. All subjects gave written informed consent in accordance with the Declaration of Helsinki.

## Author Contributions

BG and KD designed the experiment. BG and AS collected the data. TS, SM, and KD analyzed the data and wrote the manuscript.

### Conflict of Interest

The authors declare that the research was conducted in the absence of any commercial or financial relationships that could be construed as a potential conflict of interest.

## Footnotes

^1^Out of the 17 subjects, two had only high-confidence trials and hence were not considered. Out of the 15 remaining, eight were found to be significantly more affected by the cues than the rest.
