# Supplementary material for: How Our Perception and Confidence Are Altered Using Decision Cues
Source: Front Neurosci. 2020 Jan 14;13:1371. doi: 10.3389/fnins.2019.01371 (PMC6971401; doi:10.3389/fnins.2019.01371)
Supplement: Supplementary file 1 [file Data_Sheet_1.PDF]

Supplementary Information

Calculation of Cumulative Distribution Function

The cdf $G(\cdot)$ of the shifted gamma distribution (equation (1)) is given by $G(y) = 0,$ if $y \leqslant 1$, otherwise,

$$\begin{aligned} G(y) &= \int_{-\infty}^{y} \frac{\beta^\alpha}{\Gamma(\alpha)} (x-1)^{(\alpha-1)} e^{-\beta(x-1)} I_{[1,\infty)}(x)\, dx \\ &= \int_{1}^{y} \frac{\beta^\alpha}{\Gamma(\alpha)} (x-1)^{(\alpha-1)} e^{-\beta(x-1)}\, dx \\ &= \int_{0}^{y-1} \frac{\beta^\alpha}{\Gamma(\alpha)} (z)^{(\alpha-1)} e^{-\beta(z)}\, dz \\ &= H\left(y-1, \alpha, \frac{1}{\beta}\right). \end{aligned}$$

The cdf $NG(\cdot)$ of the negative gamma distribution (equation (2)) for $y \leqslant L$ is given by

$$\begin{aligned} NG(y) &= \int_{-\infty}^{y} \frac{\beta^\alpha}{\Gamma(\alpha)} (L-x)^{(\alpha-1)} e^{-\beta(L-x)} I_{(-\infty,L]}(x)\, dx \\ &= \int_{L-y}^{\infty} \frac{\beta^\alpha}{\Gamma(\alpha)} z^{(\alpha-1)} e^{-\beta(z)}\, dz \\ &= 1 - H\left(L-y, \alpha, \frac{1}{\beta}\right), \end{aligned}$$

and $NG(y) = 1$, otherwise. Here

$$I_A(x) = \begin{cases} 1 & \text{if } x \in A, \\ 0 & \text{otherwise,} \end{cases}$$

and $H(\cdot, \alpha, \frac{1}{\beta})$ is the cdf of the standard gamma distribution with shape parameter α and scale parameter $1/\beta$.

Calculation of Mode

It can be easily shown that the mode of the shifted gamma distribution is given by

$$M_g = 1 + \frac{\alpha - 1}{\beta}.$$

For finding the mode of the negative gamma distribution we start by taking the logarithm of its density (equation (2)),

$$\ln(ng(x)) = \alpha \ln(\beta) - \ln(\Gamma(\alpha)) + (\alpha - 1)\ln(L-y) - \beta(L-y).$$

Differentiating with respect to x and equating to zero, we get the mode to be

$$L - \frac{\alpha - 1}{\beta}.$$

Calculation of MLE of the parameters of the proposed mixture models

The calculation of MLE of the parameters based on EM algorithm for the mixture of a shifted gamma and a negative gamma model (equation [5]) is demonstrated here. Suppose $X_1, \ldots, X_n$ be i.i.d. observations from

$$f(x) = p\ g_{\alpha_1,\beta_1}(x) + (1-p)\ ng_{\alpha_2,\beta_2}(x).$$

We define an auxiliary variable Y_i such that

$$Y_i = \begin{cases} 1 & \text{if } X_i \sim g_{\alpha_1,\beta_1}(x), \\ 0 & \text{if } X_i \sim ng_{\alpha_2,\beta_2}(x). \end{cases}$$

So the complete likelihood and complete log-likelihood are given by

$$L = \prod_{i=1}^{n} [p\, g_{\alpha_1,\beta_1}(x_i)]^{y_i} [(1-p)\, ng_{\alpha_2,\beta_2}(x_i)]^{(1-y_i)} \text{ and}$$

$$l = \sum_{i=1}^{n} [y_i \ln(p\, g_{\alpha_1,\beta_1}(x_i)) + (1-y_i)\ln((1-p)\, ng_{\alpha_2,\beta_2}(x_i))]\,,$$

respectively. The calculations are done using E-step and M-step.

E-step

y_is' are replaced with their conditional expected values

$$\begin{aligned} \widehat{Y_i} &:= E(Y_i|X_i = x_i) \\ &= \frac{p\, g_{\alpha_1,\beta_1}(x)}{p\, g_{\alpha_1,\beta_1}(x_i) + (1-p)\, ng_{\alpha_2,\beta_2}(x_i)}. \end{aligned}$$

M-step

The MLE of p is obtained by differentiating l with respect to p and replacing the unobserved y_i by $\widehat{y_i}$ as

$$\hat{p} = \frac{\sum_{i=1}^{n} \widehat{y_i}}{n}.$$

Differentiating l with respect to α_1 and β_1, respectively, and replacing the unobserved y_i by $\widehat{y_i}$, we obtain

$$\frac{\Gamma'(\alpha_1)}{\Gamma(\alpha_1)} - \ln(\alpha_1) = \ln C_1 + \frac{\sum_{i=1}^{n} \widehat{y_i} \ln(x_i - 1)}{\sum_{i=1}^{n} \widehat{y_i}}, \tag{7}$$

and $\beta_1 = \alpha_1\, C_1$, where $\Gamma(\cdot)$ is the gamma function and $\Gamma'(\cdot)$ is it's derivative and

$$C_1 = \frac{\sum_{i=1}^{n} \widehat{y_i}}{\sum_{i=1}^{n} \widehat{y_i}(x_i - 1)}.$$

Notice that the closed form solution for the MLE of shape parameter α_1 is not tractable. So we use Newton-Raphson technique for getting the numerical solution of the equation (7). Once the MLE of α_1 is obtained as $\widehat{\alpha}_1$ using numerical technique, MLE of β_1, $\widehat{\beta}_1$, is obtained by replacing α_1 with $\widehat{\alpha}_1$ in the equation $\beta_1 = \alpha_1\, C_1$. Similarly, we differentiate l with respect to α_2 and β_2, respectively, and replace the the unobserved y_i's by $\widehat{y_i}$'s to get

$$\ln \alpha_2 - \frac{\Gamma'(\alpha_2)}{\Gamma(\alpha_2)} = \ln \frac{1}{C_2} - \frac{\sum_{i=1}^{n}(1-\widehat{y_i}) \ln(L - x_i)}{\sum_{i=1}^{n}(1-\widehat{y_i})} \tag{8}$$

and $\beta_2 = \alpha_2 C_2$, where

$$C_2 = \frac{\sum_{i=1}^{n}(1-\widehat{y_i})}{\sum_{i=1}^{n}(1-\widehat{y_i})(L - x_i)}.$$

As above, Newton-Raphson technique is employed to get MLE of α_2 as $\widehat{\alpha}_2$ and once it is obtained, the MLE of β_2 is found by replacing α_2 with $\widehat{\alpha}_2$ in the equation $\beta_2 = \alpha_2 C_2$. We start with an initial guess for the parameters $(\alpha_1, \beta_1, \alpha_2, \beta_2)$ and p and then follow the E-step and M-step, iteratively, until convergence.

For the other models given by equations (3) and (4), similar steps as described above have been followed with little modifications. Hence detailed calculations for the other models are omitted here.

One Sample Hypothesis Testing using Bootstrap

Suppose we want to test the null hypothesis (H_0) about a parameter θ of the distribution F based on a random sample $x_1, \ldots, x_n$. Further assume that the statistical test is done based on a test statistic T, measuring the discrepancy between the data and the null hypothesis, such that large values of T indicating evidence against H_0. Let the observed value of statistic be given by t. Then the achieved significance level is defined as

$$\text{ASL} = P(T \geqslant t | H_0).$$

We estimate the ASL using bootstrap resampling technique. Small value of ASL shows the evidence against the null hypothesis.

Table S1: Cases per subject where the proposed model was rejected using KS test (using Holm-Bonferroni correction) with FWER 0.05

Subject	p-value	k_1	k_2
1	0.0011	10	10
2	—	—	—
3	0.0000<br>0.0000<br>0.0384	10<br>1<br>10	10<br>1<br>5
4	0.0000<br>0.0000<br>0.0143	10<br>1<br>1	10<br>1<br>10
5	—	—	—
6	—	—	—
7	0.0011	1	1
8	—	—	—
9	—	—	—
10	0.0000<br>0.0004	10<br>5	10<br>5
11	0.0000<br>0.0000	1<br>10	1<br>10
12	—	—	—
13	—	—	—
14	—	—	—
15	—	—	—
16	0.0000<br>0.0000	1<br>10	1<br>10
17	—	—	—

The subjects are marked as '—' when the KS test failed to reject any of its cases. Clearly in more than 96% of the cases, our proposed models are accepted.

Table S2: Mean prediction error rate across subjects using 10-fold cross validation

k_1 \ k_2	1	5	10
1	0.009	0.0060	0.0077
2	0.0119	0.0086	0.0042
3	0.0055	0.0037	0
4	0	0.0309	0.0167
5	0.00625	0	0.0309
6	0.0077	0.0026	0.0179
7	0.0104	0.0073	0.0125
8	0.0370	0.030	0.0037
9	0.0214	0.0119	0.0048
10	0.0096	0.0026	0.0067

As evident from the table, the mean prediction error rate never exceeds 5%.

Table S3: Median of likelihood ratio between the proposed model and the Gaussian mixture model across subjects

k_1 \ k_2	1	5	10
1	173728.1573	3.3977	6.6146
2	2.7082	2.8670	4.6755
3	2.5848	1.6723	1.5284
4	1.0451	1.9498	1.4990
5	1.4201	**0.9932**	2.0759
6	1.2763	1.3454	1.3324
7	1.0303	**0.9708**	1.7591
8	1.0961	1.4150	1.1443
9	2.7505	1.8096	1.5784
10	28.7567	8.0835	288.6886

In all but 2 (marked in bold) cases, our model surpasses the Gaussian mixture model by a clear margin.

Table S4: Approximate achieved significance level (ASL) for testing the shift from individual decision across the subjects

k_1 \ k_2	1	5	10
1	**0.091**	0	0
2	0	0	0
3	0	0	0
4	0	0	0.003
5	0	0.005	0
6	0	0.003	0
7	0.001	0.001	0
8	0	0	0.002
9	0	0	0.003
10	0	0	0

Lower the ASL more the evidence against the null hypothesis that there is no shift from the individual decision under the influence of a social cue. It is clear that in all but one case (marked in bold), the ASL is less than 0.05.

Table S5: Proportion of decisions between k_1 and k_2 under the fitted model, for $k_1 \in \{3, 4, 5\}$ and $k_2 = 1$

sub \ k_1	3	4	5
1	0.6016	—	—
2	—	—	—
3	—	—	—
4	—	—	0.7984
5	0.4462	0.1880	0.3729
6	0.1731	0.4863	0.5574
7	—	0.6340	0.3930
8	—	—	0.4038
9	0.3790	0.5146	0.6252
10	—	—	0.5781
11	—	—	0.2888
12	0.5393	0.4024	—
13	0.7808	—	0.9219
14	0.3700	0.5984	0.6185
15	0.3991	0.4726	0.3306
16	—	—	0.4002
17	0.6201	—	—

The cases marked '—' were not considered due to very few observations for that (k_1, k_2) pair. In all other cases, there was the existence of a mode in the direction of k_2.

Table S6: Proportion of decisions between k_1 and k_2 under the fitted model, for $k_1 \in \{6, 7, 8\}$ and $k_2 = 10$

sub \ k_1	6	7	8
1	—	—	0.6635
2	—	—	—
3	—	—	—
4	0.8245	—	—
5	0.6108	0.5679	0.3386
6	0.7428	0.5922	0.3721
7	0.7776	0.7090	—
8	0.4759	—	0.6070
9	0.6512	0.4618	0.2916
10	0.6248	—	—
11	0.5959	—	—
12	0.5634	0.6094	0.6686
13	0.9534	0.8441	0.8166
14	0.1802	—	—
15	0.7409	0.7214	0.6222
16	—	—	—
17	0.7682	0.7024	0.6154

The cases marked '—' were not considered due to very few observations for that (k_1, k_2) pair. In all other cases, there was the existence of a mode in the direction of k_2.

Table S7: Proportion of decisions between k_1 and k_2 under the fitted model, for $k_1 \in \{3, 4, 5\}$ and $k_2 = 10$

sub \ k_1	6	7	8
1	0.7293	—	—
2	—	—	—
3	—	—	—
4	—	—	0.6540
5	0.6978	0.7478	0.6376
6	0.8773	0.6774	0.4338
7	—	0.0973	0.6147
8	—	—	0.6258
9	0.5831	0.7430	0.5733
10	—	—	0.7455
11	—	—	0.5767
12	0.5526	0.5739	—
13	0.7632	—	0.9073
14	0.7670	0.5751	0.2323
15	0.7586	0.6120	0.7291
16	—	—	0.4662
17	0.4062	—	—

The cases marked '—' were not considered due to very few observations for that (k_1, k_2) pair. In all other cases, there was the existence of a mode in the direction of k_2.

Table S8: Proportion of decisions between k_1 and k_2 under the fitted model, for $k_1 \in \{6, 7, 8\}$ and $k_2 = 1$

sub \ k_1	6	7	8
1	—	—	0.5862
2	—	—	—
3	—	—	—
4	0.4117	—	—
5	0.5217	0.4926	0.3471
6	0.3266	0.5007	0.5406
7	0.4153	0.4884	—
8	0.4310	—	0.2739
9	0.5610	0.6997	0.6866
10	0.5157	—	—
11	0.3344	—	—
12	0.6305	0.6511	0.7581
13	0.8309	0.6973	0.5820
14	0.7860	—	—
15	0.3965	0.4116	0.4890
16	—	—	—
17	0.4541	0.4347	0.4451

The cases marked '—' were not considered due to very few observations for that (k_1, k_2) pair. In all other cases, there was the existence of a mode in the direction of k_2.

Table S9: Average proportions of decisions within the region [1,6] when the individual choice was face, given $k_2 = 1$ vs $k_2 = 5$

sub \ k_2	1	5
1	**0.7511**	**0.6839**
2	**0.9983**	**0.7982**
3	**0.9270**	**0.8535**
4	**0.8141**	**0.6150**
5	0.7853	0.7983
6	**0.8001**	**0.7915**
7	0.7163	0.7931
8	**0.8189**	**0.7981**
9	**0.7934**	**0.6938**
10	**0.7791**	**0.6644**
11	0.6761	0.6955
12	**0.8485**	**0.7794**
13	**0.9784**	**0.8064**
14	0.9071	0.9145
15	0.7771	0.8108
16	**0.6699**	**0.6773**
17	**0.9376**	**0.9216**

The subjects whose proportion of decisions increased in case of 'FF' cue relative to 'FC/CF' are marked in bold.

Table S10: Average proportions of decisions within the region [7,11] when the individual choice was car, given $k_2 = 10$ vs $k_2 = 5$

sub \ k_2	10	5
1	**0.7802**	**0.6995**
2	**0.9909**	**0.7918**
3	**0.9483**	**0.8235**
4	**0.8390**	**0.6934**
5	**0.6971**	**0.6330**
6	0.8142	0.8359
7	**0.8587**	**0.8200**
8	0.7937	0.8131
9	**0.6047**	**0.4368**
10	**0.7343**	**0.5576**
11	**0.7627**	**0.7436**
12	**0.8135**	**0.7581**
13	**0.9291**	**0.8744**
14	**0.1802**	**0.1543**
15	**0.8683**	**0.7740**
16	0.8444	0.8461
17	**0.8828**	**0.7817**

The subjects whose proportion of decisions increased in case of ‘CC’ cue relative to ‘FC/CF’ are marked in bold.

Table S11: Approximate achieved significance level (ASL) for testing the cross-over from individual decision towards contradictory social cue

k_1 \ k_2	1	5	10
1			0
2			0
3			0
4			0
5			0
6	0		
7	0		
8	**0.1220**		
9	0		
10	0		

Lower the ASL more the evidence against the null hypothesis that the proportion of subjects having a crossover is not more than 0.5. It is clear that in all but one case (marked in bold), the ASL is less than 0.05.

Table S12: Cross-over area under the influence of conflicting cue ‘CC’

sub \ (k_1, k_2)	(1,10)	(2,10)	(3,10)	(4,10)	(5,10)
1	0.3251	0.6484	0.6529	—	—
2	0.1526	0.2324	—	—	—
3	0.2711	0.4388	—	—	—
4	0.5984	—	—	—	0.6361
5	nco	0.1659	0.1983	0.3208	0.4485
6	—	0.1442	0.2044	0.2887	0.3838
7	0.0850	0.1460	—	0.0911	0.5606
8	0.0348	0.0800	—	—	0.5501
9	0.2207	0.3162	0.2630	0.4167	0.5075
10	0.4110	0.6277	—	—	0.7041
11	0.1604	—	—	—	0.5345
12	0.0277	0.1215	0.3206	0.4937	—
13	0.2497	0.2171	0.6439	—	0.8438
14	nco	nco	0.1953	nco	0.1381
15	0.0719	0.1199	0.2166	0.4089	0.6591
16	0.1540	—	—	—	0.4651
17	0.0357	0.0633	0.1971	—	—

The cases marked ‘—’ were not considered due to very few observations for that (k_1, k_2) pair. If there is a cross-over we estimate the proportion of decisions in the direction of the social cue ‘CC’, i.e., $k_2 = 10$, by the area under the fitted density between 6 and 11. No cross over cases are marked as ‘noc’.

Table S13: Cross-over area under the influence of conflicting cue 'FF'

(k_1, k_2) / sub	(6,1)	(7,1)	(8,1)	(9,1)	(10,1)
1	—	—	0.4928	0.4982	0.3434
2	—	—	—	0.5388	0.3926
3	—	—	—	—	0.5247
4	0.4117	—	—	—	0.5073
5	0.5217	0.3410	0.1830	—	—
6	0.3266	0.2361	nco	—	—
7	0.4153	0.4058	—	—	0.1602
8	0.4310	—	nco	0.2538	0.1938
9	0.5610	0.5466	0.5863	—	—
10	0.5157	—	—	0.7328	0.5577
11	0.3344	—	—	—	0.1281
12	0.6305	0.5799	0.5918	0.3035	0.0596
13	0.8309	0.6385	0.2783	—	0.5520
14	0.7860	—	—	—	—
15	0.3965	0.2553	0.1781	0.3729	nco
16	—	—	—	—	0.2214
17	0.4541	nco	nco	0.0825	nco

The cases marked '—' were not considered due to very few observations for that (k_1, k_2) pair. If there is a cross-over we estimate the proportion of decisions in the direction of the social cue 'FF', i.e., $k_2 = 1$, by the area under the fitted density between 1 and 6. No cross over cases are marked as 'noc'.

Table S14: Comparison of cross over areas under conflicting and concurring cues

Conf−Conc	p-value
$(1, 10) - (1, 1)$	0.0000
$(2, 10) - (2, 1)$	0.0004
$(3, 10) - (3, 1)$	0.0039
$(4, 10) - (4, 1)$	0.2344
$(5, 10) - (5, 1)$	0.0005
$(6, 1) - (6, 10)$	0.0017
$(7, 1) - (7, 10)$	0.0078
$(8, 1) - (8, 10)$	0.0156
$(9, 1) - (9, 10)$	0.0078
$(10, 1) - (10, 10)$	0.0005

p-values of the right-tailed paired Wilcoxon signed rank test for the null hypothesis that A-B has zero median, where A contains the cross-over areas across the subjects under conflicting cue and B contains the cross-over areas across the subjects under concurring cue for the same value of k_1 at 5% significance level. It is evident that the cross-over is significantly more for conflicting cues.

Table S15: Two-way anova table

Source	SS	df	MS	F	Prob>F
Columns	0.00006	1	0.00006	0.04	0.8414
Rows	0.01006	3	0.00335	2.1	0.1031
Interaction	0.000143	3	0.00014	0.09	0.9659
Error	0.20419	128	0.0016		
Total	0.21475	135			

Columns represent face/car, rows represent the 4 conditions (no comment, FF, FC/CF, CC) with 17 repetitions each. The differences are all insignificant as evident from the table.

Table S16: Significant voxels from statistical non-parametric test for concurring < conflicting using sLORETA during 200-250 ms post stimulus onset.

X(MNI)	Y(MNI)	Z(MNI)	VoxelValue	Brodmann area	Lobe	Structure
-40	55	0	-2.65E+00	10	Frontal Lobe	Middle Frontal Gyrus
-50	15	10	-2.59E+00	44	Frontal Lobe	Precentral Gyrus
-45	15	10	-2.56E+00	44	Frontal Lobe	Precentral Gyrus
-55	20	10	-2.54E+00	45	Frontal Lobe	Inferior Frontal Gyrus
-45	50	5	-2.50E+00	10	Frontal Lobe	Middle Frontal Gyrus
-40	50	5	-2.50E+00	10	Frontal Lobe	Inferior Frontal Gyrus
-55	15	10	-2.50E+00	44	Frontal Lobe	Precentral Gyrus
-45	50	0	-2.47E+00	10	Frontal Lobe	Inferior Frontal Gyrus
-50	20	10	-2.46E+00	45	Frontal Lobe	Inferior Frontal Gyrus
-40	55	5	-2.44E+00	10	Frontal Lobe	Inferior Frontal Gyrus
-40	45	0	-2.44E+00	10	Frontal Lobe	Sub-Gyral
-40	55	-5	-2.43E+00	10	Frontal Lobe	Middle Frontal Gyrus
-45	10	10	-2.41E+00	44	Frontal Lobe	Precentral Gyrus

Table S17: Significant voxels from statistical non-parametric test for concurring < conflicting using sLORETA during 250-300 ms post stimulus onset.

X(MNI)	Y(MNI)	Z(MNI)	VoxelValue	Brodmann area	Lobe	Structure
-40	55	0	-2.41E+00	10	Frontal Lobe	Middle Frontal Gyrus
-50	15	10	-2.30E+00	44	Frontal Lobe	Precentral Gyrus
-40	45	0	-2.26E+00	10	Frontal Lobe	Sub-Gyral
-45	15	10	-2.26E+00	44	Frontal Lobe	Precentral Gyrus
-40	50	5	-2.25E+00	10	Frontal Lobe	Inferior Frontal Gyrus
-45	50	5	-2.24E+00	10	Frontal Lobe	Middle Frontal Gyrus
-45	50	0	-2.23E+00	10	Frontal Lobe	Inferior Frontal Gyrus
-55	15	10	-2.22E+00	44	Frontal Lobe	Precentral Gyrus
-55	20	10	-2.21E+00	45	Frontal Lobe	Inferior Frontal Gyrus
-45	10	10	-2.17E+00	44	Frontal Lobe	Precentral Gyrus
